# P-cadherin overexpression is associated with early transformation of the Fallopian tube epithelium and aggressiveness of tubo-ovarian high-grade serous carcinoma

**DOI:** 10.1007/s00428-025-04104-7

**Published:** 2025-05-05

**Authors:** Rita Canário, Ana Sofia Ribeiro, Inês Morgado, Ana Peixoto, Ana Barbosa, Catarina Santos, Nuno Mendes, Paula Lopes, Paula Monteiro, Ricardo Coelho, Francis Jacob, Viola Heinzelmann-Schwarz, Sara Ricardo, Manuel R. Teixeira, Carla Bartosch, Joana Paredes

**Affiliations:** 1 Graduate Program in Areas of Basic and Applied Biology (GABBA), School of Medicine and Biomedical Sciences (ICBAS), University of Porto, Porto, Portugal; 2 Cancer Metastasis, i3S–Institute for Research and Innovation in Health, University of Porto, Porto, Portugal; 3 Cancer Biology and Epigenetics Group, Research Center–Portuguese Oncology Institute of Porto (CI-IPO-Porto)/RISE@CI-IPOP (Health Research Network), Portuguese Oncology Institute of Porto/Porto. Comprehensive Cancer Center Raquel Seruca (P.CCC Raquel Seruca), Porto, Portugal; 4 Cancer Genetics Group, Research Center–Portuguese Oncology Institute of Porto (CI-IPO-Porto)/RISE@CI-IPOP (Health Research Network), Portuguese Oncology Institute of Porto/Porto. Comprehensive Cancer Center Raquel Seruca (P.CCC Raquel Seruca), Porto, Portugal; 5 Department of Laboratory Genetics, Portuguese Oncology Institute of Porto (IPO-Porto)/Porto. Comprehensive Cancer Center Raquel Seruca (P.CCC Raquel Seruca), Porto, Portugal; 6 Histology and Electron Microscopy, i3S–Institute for Research and Innovation in Health, University of Porto, Porto, Portugal; 7 Pathology Department, Portuguese Oncology Institute of Porto (IPO-Porto)/Porto. Comprehensive Cancer Center Raquel Seruca (P.CCC Raquel Seruca) , Porto, Portugal; 8 Ovarian Cancer Research, Department of Biomedicine, University Hospital Basel and University of Basel, 4031 Basel, Switzerland; 9 Ageing and Stress Group, i3S–Institute for Research and Innovation in Health,, University of Porto, Porto, Portugal; 10 Associate Laboratory I4HB, Institute for Health and Bioeconomy, University Institute of Health Sciences—CESPU, Gandra, Portugal; 11 UCIBIO—Applied Molecular Biosciences Unit, Toxicologic Pathology Research Laboratory, University Institute of Health Sciences (1H-TOXRUN, IUCS-CESPU), Gandra, Portugal; 12 Pathology and Molecular Immunology Department, School of Medicine and Biomedical Sciences (ICBAS), University of Porto, Porto, Portugal; 13 FMUP—Faculty of Medicine, University of Porto, Porto, Portugal

**Keywords:** P-cadherin, *CDH3*, Epithelial-to-mesenchymal plasticity (EMP), High-grade serous carcinoma, Homologous recombination

## Abstract

Tubo-ovarian high-grade serous carcinoma (HGSC) with proficient homologous recombination (HR) DNA repair (HRP) accounts for approximately 50% of cases and is associated with platinum-resistance and poor prognosis. We hypothesize that the acquisition of hybrid phenotypes displaying both epithelial and mesenchymal (E/M) features may be involved in the malignant transformation and tumour dissemination in this subgroup. Therefore, we analysed, by digital pathology, the expression and prognostic significance of 3 classic cadherins (E-cadherin, epithelial marker; N-cadherin, mesenchymal marker; and P-cadherin, candidate marker of hybrid E/M) in 577 formalin-fixed paraffin-embedded human samples representing the putative stepwise serous carcinogenesis in the Fallopian tube epithelium (FTE). We observed a non-canonical N-to-P-cadherin switch along the carcinogenic progression, with a statistically significant overexpression of P-cadherin in pre-malignant and malignant samples, compared to the control FTE. Interestingly, this overexpression was most pronounced in precursor lesions and HGSC cells from malignant ascites. Tumours with high P-cadherin expression were significantly associated with worse overall survival, especially in the subgroup without *BRCA1/2* mutations. Transient P-cadherin knock-down resulted in in vitro significant reduction of functional hybrid E/M hallmarks, namely decreased *anoikis* resistance, reduced collective migration and invasion in a representative platinum-resistant HRP cell line. Taken together, our results suggest that P-cadherin overexpression is an early event in the serous carcinogenesis and may be involved in hybrid E/M activation in HRP-HGSC, further supporting this adhesion molecule as a promising biomarker for this poor prognostic subgroup.

**Supplementary information:**

The online version contains supplementary material available at 10.1007/s00428-025-04104-7.

## Introduction

High-grade serous carcinoma (HGSC) is the most common and deadliest histologic subtype of tubo-ovarian carcinoma [1]. Its aggressive biological nature often leads to diagnoses in advanced stages with widespread peritoneal metastasis and malignant ascites, impacting negatively on patient prognosis [2]. Approximately 50% of cases exhibit homologous recombination (HR) DNA repair deficiency (HRD), primarily due to genetic and epigenetic alterations of HR genes, most commonly in *BRCA1* and *BRCA2* [3]. Tumours with proficient HR (HRP) are associated with platinum-resistance and worse prognosis [4]. Therefore, identifying new biomarkers for this subgroup is an urgent and unmet clinical need.

Epithelial-mesenchymal plasticity (EMP) has been associated with some functional hallmarks that are crucial for tumour promotion and dissemination, such as collective cell migration, resistance to programmed cell death upon basement membrane or extracellular matrix detachment (*anoikis*) and stem-like properties [5–7]. Indeed, ovarian cancer cells displaying both epithelial and mesenchymal (E/M) features (hybrid E/M) have been associated with increased stemness, chemo-resistance and poor prognosis [8–10]. Interestingly, serous tubal intraepithelial carcinomas (STICs)—the precursor lesions found in the fimbriae of the Fallopian tube epithelium (FTE)—already display morphologic features suggesting the activation of EMP, namely loss of cell polarity with irregular luminal borders, intraepithelial fractures and exfoliation [11–13]. Moreover, tumour budding and free-floating clusters are frequently observed in HGSC [11, 12]. However, whether the acquisition of hybrid E/M features in FTE cells contributes to the malignant transformation and dissemination of HGSC, or if it could serve as a potential prognostic or predictive biomarker, remains to be determined.

In this work, we investigated the EMP dynamics during the process of malignant transformation and dissemination of HGSC. To this end, we analysed the expression and prognostic significance of canonical epithelial-to-mesenchymal transition cell surface markers—E-cadherin (epithelial marker) and N-cadherin (mesenchymal marker)—along with P-cadherin, a candidate marker of hybrid E/M phenotypes [14]. This analysis was conducted using human samples representing the stepwise progression of serous carcinogenesis, complemented by functional in vitro assays performed on ovarian cancer cell lines.

## Material and methods

### Patients and tissue samples

#### Oporto series

Five cohorts of patients were selected to investigate the putative stepwise serous carcinogenesis using archival material routinely collected (Fig. S1). In brief, HGSC primary tumours and solid metastasis were obtained from the expansion of a previously reported case series (1/2016–6/2020) [15], further enriched with stratified sampling of HGSC cases harbouring germline *BRCA1/2* mutations (2001–2015), to ensure the representativeness of HRD samples (Table 1 and Fig. S2). A representative formalin-fixed paraffin-embedded (FFPE) tissue block demonstrating distinct morphological patterns of the primary tumour, obtained from either the Fallopian tube or ovary, was selected. Additionally, a paired peritoneal metastasis sample was included whenever available. Metastatic samples from other sites were assessed only when peritoneal specimens were unavailable. The median follow-up time for survivors was 118.5 months (95% confidence interval: 74.4–162.5). As of the data censoring date (November 2023), 45 patients (46.9%) had died from the disease, 7 patients (7.3%) had died from other causes, 37 patients (38.5%) were alive with no evidence of disease, and 7 patients (7.3%) were alive with disease. Ascitic fluid from HGSC patients was obtained within the framework of a partner project [16]. Fallopian tube samples were collected from patients who underwent salpingectomy between 2012 and 2017, with no malignancy identified in the surgical tissue (Fig S3). These samples were used as the control FTE, with 59.6% (*n* = 59) being from individuals harbouring pathogenic germline *BRCA1/2* mutations (high-risk). This study was approved by the Ethics Committee of IPO-Porto (CES. Ref. 91/018 and Ref.92R1/019) and exempted from informed consent due to the retrospective analysis of archival material.

**Table 1:** Clinicopathologic baseline features between Oporto and Basel series

Clinical features	Oporto series *N* = *96 patients*	Basel series *N* = *116 patients*	***Group comparisons*** (Pearson *X*^2^)
**Age at diagnosis** in years, mean [min, max]	59.1 [36, 83]	63.8 [37, 91]	*p* = 0.002**
**Staging (FIGO)**			
FIGO I/II	14 (14.6%)	5 (4.3%)	*p* = 0.009**
FIGO III/IV	80 (83.3%)	109 (94.0%)	
Incomplete staging (≧FIGO IC)	2 (2.1%)	2 (1.7%)	
**Homologous repair deficiency** (germline or somatic pathogenic mutations in HR genes)	**36 (37.5%)**	**14 (12.1%)**	*p* < 0.001*** (HRD vs HRP)
*BRCA1*	19 (19.8%)	9 (7.8%)	
* BRCA2*	13 (13.5%)	4 (3.4%)	
* BRCA1* + *BRCA2*	0 (0%)	1 (0.9%)	
* RAD51D*	3 (3.1%)	0 (0%)	
*ATM*	1 (1.0%)	0 (0%)	
**Cytoreduction **(*n*, %)	**87 (90.6%)**	**116 (100%)**	*p* > *0.05* (complete vs incomplete debulking)
Of which, complete debulking (macroscopic tumour ≦1 cm)	36 (48.0%)	36 (31.0%)	
**Chemotherapy**	**92 (95.8%)**	**95 (81.9%)**	*p* = 0.018*
Adjuvant	60 (62.5%)	68 (58.6%)	
Neoadjuvant	24 (25.0%)	26 (22.4%)	
Palliative	8 (8.3%)	1 (0.9%)	
No systemic treatment	4 (4.2%)	0 (0%)	
Unknown	0 (0%)	21 (18.1%)	
**Platinum sensitivity at baseline**		NA	–
Sensitive (PD ≧ 6 months after completion of 1 st line CT)	74 (77.1%)		
Resistant (PD < 6 months after completion of 1 st line CT)	9 (9.4%)		
Unknown	13 (13.5%)		
**Maintenance treatment (regardless of the line)**	**39 (40.6%)**	NA	–
PARP inhibitors	20 (20.8%)		
Bevacizumab	19 (19.8%)		
**Hyperthermic intraperitoneal chemotherapy treatment**	**11 (11.5%)**	NA	–
**Cadherin immunoexpression in primary tumours **(H-scores median/IQR)	**80 samples**	**88 samples**	
E-cadherin	283.94 (263.86–293.61)	225.06 (150.39–270.90)	*p* < 0.001***
N-cadherin	114.71 (66.65–172.57)	143.04 (104.54–207.14)	*p* = 0.003**
P-cadherin	131.27 (68.17–203.60)	173.65 (121.59–222.99)	*p* = 0.004**
**Cadherins immunoexpression in peritoneal metastases **(H-scores median/IQR)	**51 samples**	**87 samples**	
E-cadherin	280.44 (252.98–289.26)	166.07 (76.68–253.91)	p < 0.001***
N-cadherin	102.51 (53.95–167.52)	120.93 (95.29–166.49)	*p > 0.05*
P-cadherin	105.14 (50.61–199.64)	107.19 (83.35–179.00)	*p* > 0.05

#### Basel series

A HGSC validation case series was obtained from the Ovarian Cancer Research Group (Francis Jacob and Viola Heinzelmann-Schwarz), University Hospital of Basel and University of Basel, Switzerland. Samples from this TMA series were collected between 1992 and 2017 and consisted of 221 tumour-cores derived from 88 primary HGSC obtained from the Fallopian tube or ovary and 133 metastases from different sites (65.4% peritoneal metastases, of which 66 were paired with primary tumour), from 116 patients (Table 1). Only TMAs with preserved tissue available in triplicated to allow IHC staining were included. The use of this series was approved by the Swiss Medical Ethics Committee (EKNZ:2015–436 and EKNZ:2023–00988).

### Cell lines and culture

Ovarian cancer cell lines were kindly given by Professor Henrik Clausen (University of Copenhagen) (OVCAR3) and Dr. Francis Jacob (University of Basel) (OVCAR4 and BG1). Cell lines tested negative for *Mycoplasma* contamination and were authenticated using short tandem repeat profiling. Details on culture conditions are shown in Table S1.

### Antibodies and reagents

Detailed on Tables S2 & S3.

### Histology, immunostaining, and digital pathology scoring

Tissue sections were immunostained according to optimised protocols, as previously described for E-, N-, and P-cadherin [17]. The downstream immunohistochemistry (IHC) protocol for p53, Ki67, and PAX8 was performed on a fully automated BenchMark® ULTRA (Ventana, Tucson, AZ) using UltraView Universal DAB Detection Kit (Roche, Basel, Switzerland). Digitalized whole slides/TMAs were analysed using an open-source digital pathology software (QuPath®, version 0.2.3) [18]. Cases exhibiting distinct staining patterns and intensities were selected, and tumour/stromal cell areas were manually annotated to train the machine learning algorithm in QuPath®, using a random pixel classifier with an artificial neural network. Tumour cells were then annotated based on the pixel classifier and detected using the watershed cell membrane detection algorithm, relying on the diaminobenzidine (DAB) staining pattern. Slide evaluation was conducted in parallel by an experienced gynaecological pathologist (CB), blinded to clinicopathological data, using traditional pathology to validate the algorithm. After ensuring its fidelity, cells were classified based on DAB optical density mean intensity in the membrane region, with three thresholds defined according to marker expression percentiles. Following intensity cut-off determination, cadherin expression levels in tumour areas were quantified using the H-score method in QuPath®, generating a score ranging from 0 to 300 [19].

### Next-generation sequencing

Next-generation sequencing (NGS) was performed on DNA extracted from cell lines using the TruSight Hereditary Cancer panel (Illumina, Inc., San Diego, CA, USA), as previously described [20].

### siRNA transfection

Gene silencing with small interfering RNA (siRNA) was performed using a *CDH3* siRNA (siCDH3). A siRNA control with no homology to any human gene was used as a negative control (siCTRL). Transfection was carried out 24 h post-seeding using 3 μL Lipofectamine2000® (Invitrogen™, Thermo Fisher Scientific Inc) according to the manufacturer’s recommendations.

### Functional assays

#### Wound healing assay

Cells were seeded 24 h post-transfection and incubated for additional 24 h to form a complete monolayer. Then, a wound was made, and the distances between its edges were monitored every 15 min for 24 h, using a LEICA Timelapse DMI6000 microscope (Leica Microsystems, Wetzlar, Germany). Quantitative analysis was performed with ImageJ freehand selection tool.

#### Collagen type I 3D invasion assay

Transfected cells were seeded and incubated for 24 h or 48 h (BG1 or OVCAR4, respectively) to allow spheroid formation, which were further embedded into a rat tail collagen type I matrix, as described elsewhere [21] and monitored every 30 min for 24 h using a LEICA Timelapse DMI6000 microscope. Quantitative analysis of the invaded area or number of isolated invasive cells (for OVCAR4 or BG1, respectively) was performed using ImageJ.

#### Sphere formation assay

A single-cell suspension was plated at a low density in non-adherent culture conditions upon siRNA transfection, and allowed to grow for 5 days. Sphere forming efficiency (SFE) was calculated as previously described [22].

#### Cell viability assay 

Cells were treated with PrestoBlue™ reagent according to the manufacturer’s instructions, 24 h post-transfection. Fluorescence was measured at 560 nm (emission) and 590 nm (excitation), 30–45 min after incubation at the fluorescence-based reader Synergy Mx™ (BioTek Instruments, Inc., Winooski, VT, USA).

Details on experimental conditions can be found in Table S1.

### Western blot

Western blot was performed as previously described [23]. Bands in the immunoblots were quantified by densitometric analysis using Quantity One® software (Bio-Rad Inc, Hercules, California, USA).

### Statistical analysis

Continuous variables were described as mean and range. Immunoexpression (H-score) was described as median and interquartile range (IQR) and computed into categorical variables using the median cut-off (low and high expression). Group comparisons were made using the adequate parametric and non-parametric tests. Univariate analysis of progression-free survival (time from cytoreduction till progression or last follow-up) and cancer-specific survival (time from diagnosis of HGSC till death due to cancer or last follow-up) were calculated using the Kaplan–Meier and log-rank test. Multivariate analysis was performed using Cox regression, including the significant results from univariate analysis. Hazard ratios (HR) and respective 95% confidence intervals (CI) were reported. Data were analysed using IBM SPSS Statistics (version 29.0.2.0; IBM Corp, Armonk, NY) and Graph Pad Prism version 8.0 software (Graph Pad Software, San Diego, CA, USA). A *p*-value < 0.05 was considered statistically significant. Bonferroni correction was applied for multiple testing.

## Results

### P-cadherin is overexpressed in HGSC precursor lesions

**Fig. 1:**
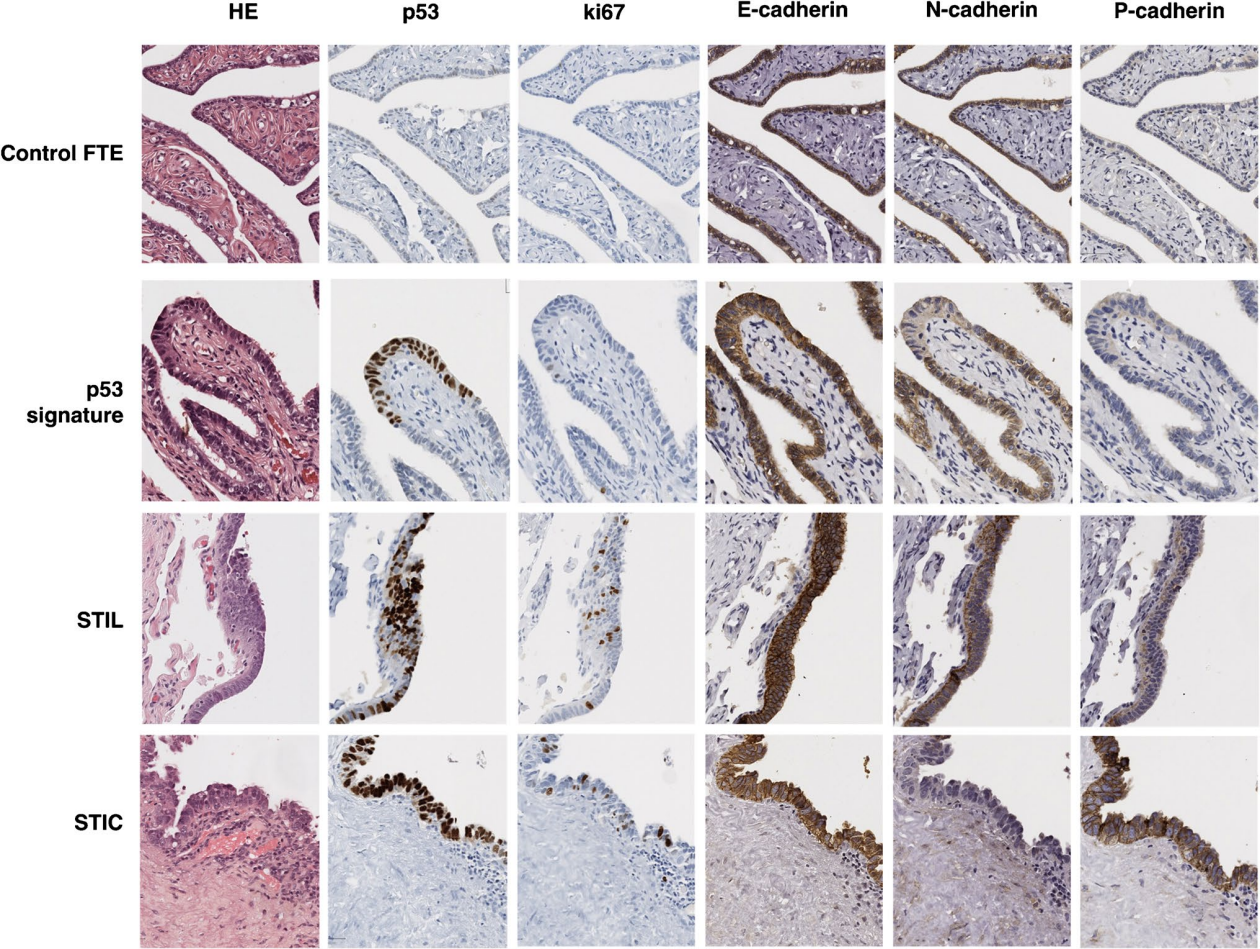
**Cadherins’ expression in control FTE and serous precursor lesions in Oporto series.** Representative histology sections of the control FTE and distinct serous precursor lesions (amplification 20x). The precursor lesions included in the evaluation of cadherin expression were identified in patients with HGSC (cohort 4) or discovered incidentally during surgery for other gynaecologic conditions (cohort 3). These lesions were diagnosed by screening the distal portion of the FTE samples according to morphological criteria, combined with immunohistochemical markers (p53, Ki67 and PAX8)

Samples from FTE of low- and high-risk cohorts were stained for E-, N- and P-cadherin. No significant differences were observed between the two cohorts and, therefore, we used the combined samples as the control FTE. E-cadherin and N-cadherin showed strong and diffuse cell membrane and cytoplasmic expression in secretory and ciliated cells, with median H-scores above 270 (Table 2, Fig. 1). In contrast, P-cadherin staining was low to very low intensity, characterised by diffuse cytoplasmic expression restricted to the secretory cells (Table 2, Fig. 1). However, P-cadherin expression was significantly higher in the FTE adjacent to STICs and/or HGSC, when compared to control FTE (Table 2, Fig. 1, and Fig. S4). Even though some significant differences in E-cadherin expression were observed between groups, overall immunoexpression scores remained very high (Table 2). No differences in expression were observed for N-cadherin (Fig. S4).

**Table 2:** Cadherin expression in Oporto series

Sample type	*N*	E-cadherin H-score			N-cadherin H-score			P-cadherin H-score		
		Median	IQR	*p-value *^a^	Median	IQR	*p-value *^a^	Median	IQR	*p-value *^a^
Control FTE	99	297.32	295.55–298.40	< *0.001*	273.61	255.94–285.40	< *0.001*	76.10	52.78–105.51	< *0.001*
Adjacent FTE	34	292.30	252.41–298.35		270.05	229.92–290.56		174.78	117.40–213.90	
p53 signatures + serous tubal intraepithelial lesions (STIL)	19	300.00	299.06–300.00		284.91	233.78–300.00		216.92	112.90–288.57	
STIC	40	299.31	296.65–299.81		287.61	227.96–298.63		276.59	215.81–296.92	
Primary tumours	80	283.94	263.86–293.61		114.71	66.65–172.57		131.27	68.17–203.60	
Solid metastasis	59	279.81	252.98–289.26		96.07	52.69–167.52		129.59	50.61–202.22	
Ascitic fluid metastasis	25	281.88	266.75–293.82		133.69	31.50–254.84		248.23	159.05–274.99	

^a^Comparisons between groups were made using Independent-samples Kruskal–Wallis test. Pairwise comparisons are provided in Fig. S4

These observations prompted us to hypothesise that P-cadherin may play a role in the early stages of serous carcinogenesis. To investigate this, we characterised cadherins’ expression in 59 serous precursor lesions from 32 patients (43% HRD). These lesions were identified by screening the fimbriated portion of available FTE samples from the Oporto series according to morphological criteria, combined with immunohistochemical markers (p53, Ki67 and PAX8) [13]. Remarkably, P-cadherin was the only cadherin whose expression was significantly increased in precursor lesions compared to both control and adjacent FTE, with diffuse staining observed in both the cytoplasm and cell membrane (Table 2, Fig. 1, and Fig. S4). Moreover, P-cadherin demonstrated a significant increase in expression during malignant transformation, progressing from p53 signatures and serous tubal intra-epithelial lesions (STILs) to STICs (Fig. 1 and Fig. S4). Collectively, these results suggest a potential role for P-cadherin in the early stages of serous carcinogenesis.

### N-to-P-cadherin switch occurs in HGSC carcinogenesis

**Fig. 2:**
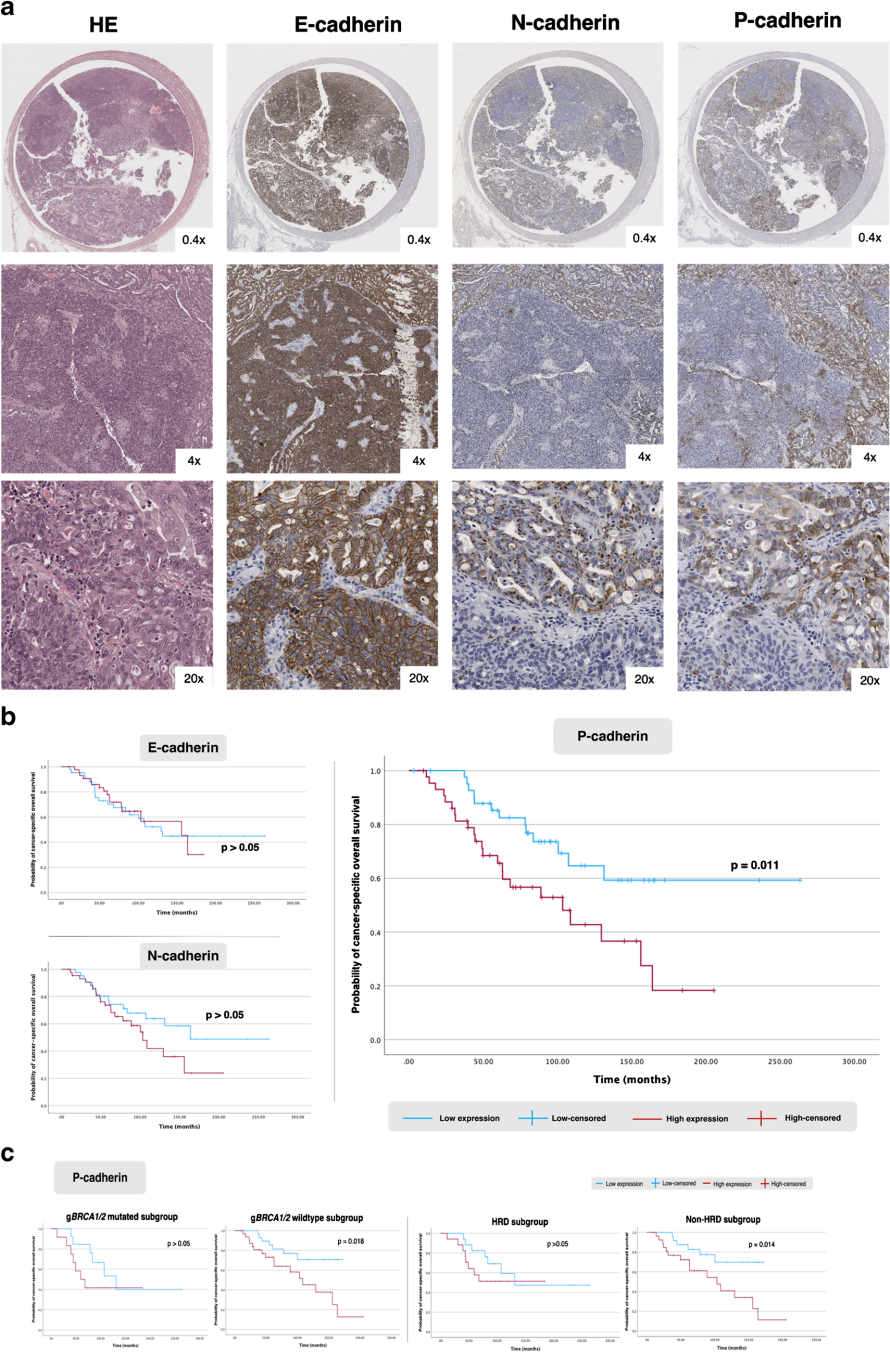
**Cadherins’ expression and prognostic significance in HGSC tumours from Oporto series. a.** Histology images of an intra-tubar HGSC staining for E-, N- and P-cadherin. Dot-like staining is notorious at 20x magnification for N-cadherin and intra-tumoral heterogeneity is evident for both N- and P-cadherin. **b.** Survival plots displaying Kaplan-Meier estimates for cancer-specific overall survival according to E-cadherin, N-cadherin and P-cadherin expression. **c.** Post-hoc subgroup analysis is shown for P-cadherin expression according to germline *BRCA1/2* (g*BRCA1/2*) and HR mutational status. The HRD subgroup includes patients with identified somatic (tumor) and/or germline mutations HR genes (Table 1), while the non-HRD subgroup comprises patients without *BRCA1/2 *mutations

To investigate the pattern of expression of the three classical cadherins in HGSC, we stained 80 primary tumours and 59 solid metastases from Oporto series (Fig. 2a). We observed that all primary tumours co-expressed the three cadherins (Table 2, Fig. S4). E-cadherin was homogeneously expressed in most tumours, with a diffuse, strong, and predominantly membrane-localised staining. This cadherin was expressed in all tumours analysed (median of positive cells: 98.0%, IQR 95.0–99.1) and exhibited the highest H-scores (Table 2). In contrast, N-cadherin and P-cadherin exhibited inter- and intra-tumoral heterogeneity, with variations in the percentage of positive cells and staining intensity (Fig. 2a, Table 2). Despite the heterogeneous expression, these proteins did not show an association with any specific architectural pattern in HGSC. P-cadherin was expressed in both the cell membrane and cytoplasm of tumour cells; but it also stained the mesothelium, Walthard’s nests and Hydatid of Morgani, when present (Fig. S5). N-cadherin was expressed predominantly in the cytoplasm, with focal dot-like staining observed in 18 cases (Fig. 2a). We then analysed metastatic implants from extra-gynaecological topography (*n* = 59), mostly peritoneal (86.4%). These samples showed similar expression patterns to primary tumours. Interestingly, the N-cadherin dot-like staining pattern, displayed in some of the primary tumours (*n* = 18), was maintained in most of the available paired metastatic implants (6 out of 9 cases). We observed a non-significant decreasing trend between primary tumour and matched peritoneal metastases for each cadherin (Fig. S6, Table 1). Remarkably, we observed similar expression patterns for the three cadherins in the Basel series (Table 1). However, there was a significant reduction in H-scores for E- and N-cadherin in peritoneal metastases, compared to matched primary tumours (Fig. S6). Similarly, E- and P-cadherin expression, but not N-cadherin, were significantly reduced in metastatic lymph nodes when compared to primary tumours. Notably, despite the statistically significant reduction in E-cadherin, the H-scores of both tumours and metastases were consistently very high across almost all tumours analysed.

Finally, we compared cadherins expression in Oporto primary tumours with control FTE, and P-cadherin was again significantly overexpressed in tumours, whereas N-cadherin was significantly downregulated (Table 2, Fig. S4). These findings collectively highlight the occurrence of a N-to-P-cadherin switch during serous carcinogenesis, reflected by the transition from the constitutively expressed N-cadherin to the *de novo* expression of P-cadherin.

### P-cadherin overexpression in HGSC is associated with shorter cancer-specific survival

To determine whether the overexpression of P-cadherin was associated with increased aggressiveness of HGSC, we analysed its association with standard clinicopathological features linked to prognosis in the Oporto series, where we had access to patients’ survival data. No differences were observed in the baseline prevalence of these features according to high or low cadherins expression (Table S4). Nevertheless, high P-cadherin expression was uniquely and significantly associated with reduced cancer-specific overall survival (Fig. 2b). In addition, a post-hoc analysis revealed that high P-cadherin H-scores were only significantly associated with shorter cancer-specific survival in the germline *BRCA1/2* wild-type and non-HRD subgroups (Fig. 2c). In the multivariate analysis, only platinum sensitivity and completeness of cytoreductive surgery, but not P-cadherin expression, emerged as independent prognostic factors (Table 3).

**Table 3:** Univariate and multivariate analysis of predictive factors associated with HGSC survival outcomes

Factor	Disease-free survival					Ovarian cancer-specific survival				
	Univariate		Multivariate			Univariate		Multivariate		
	***n***	***p-***value	HR	95% CI	***p-***value	***n***	***P-***value	HR	***95% CI***	***p-***value
Age (years)^a^										
< 58	44	0.724				45	0.401			
≥ 58	42					42				
FIGO stage (2014)										
Stage I/II	13		3.171	0.718–14.004	0.128	13				
Stage III/IV	71	0.019*				72	0.181			
*BRCA1/2 status (germline)*										
*Deleterious mutation*	24					25				
*Wildtype*	62	0.091				62	0.412			
Homologous repair status (tumour and/or germline)										
HRD	33					34				
Non-HRD	53	0.432				53	0.911			
Residual disease after cytoreduction										
< 1 cm	36		1.703	0.810–3.578	0.160	36		3.105	1.167–8.262	0.023*
≧ 1 cm	31	0.015*				31	0.018*			
Platinum sensitivity (first line)										
Resistant (PFI < 6 months)	6		45.443	5.939–347.729	< 0.0001****	6		37.872	9.571–149.853	< 0.001***
Sensitive (PFI ≧6 months)	72	< 0.001***				72	< 0.001***			
E-cadherin tumour expression^a^										
Low	42					43				
High	44	0.398				44	0.889			
N-cadherin tumour expression^a^										
Low	43					43				
High	43	0.341				44	0.149			
P-cadherin tumour expression^a^										
Low	42					43		2.215	0.899–5.455	0.084
High	44	0.733				44	0.011*			

^**a**^Median value used as cut-off

### HGSC peritoneal effusions show enriched P-cadherin expression

**Fig. 3:**
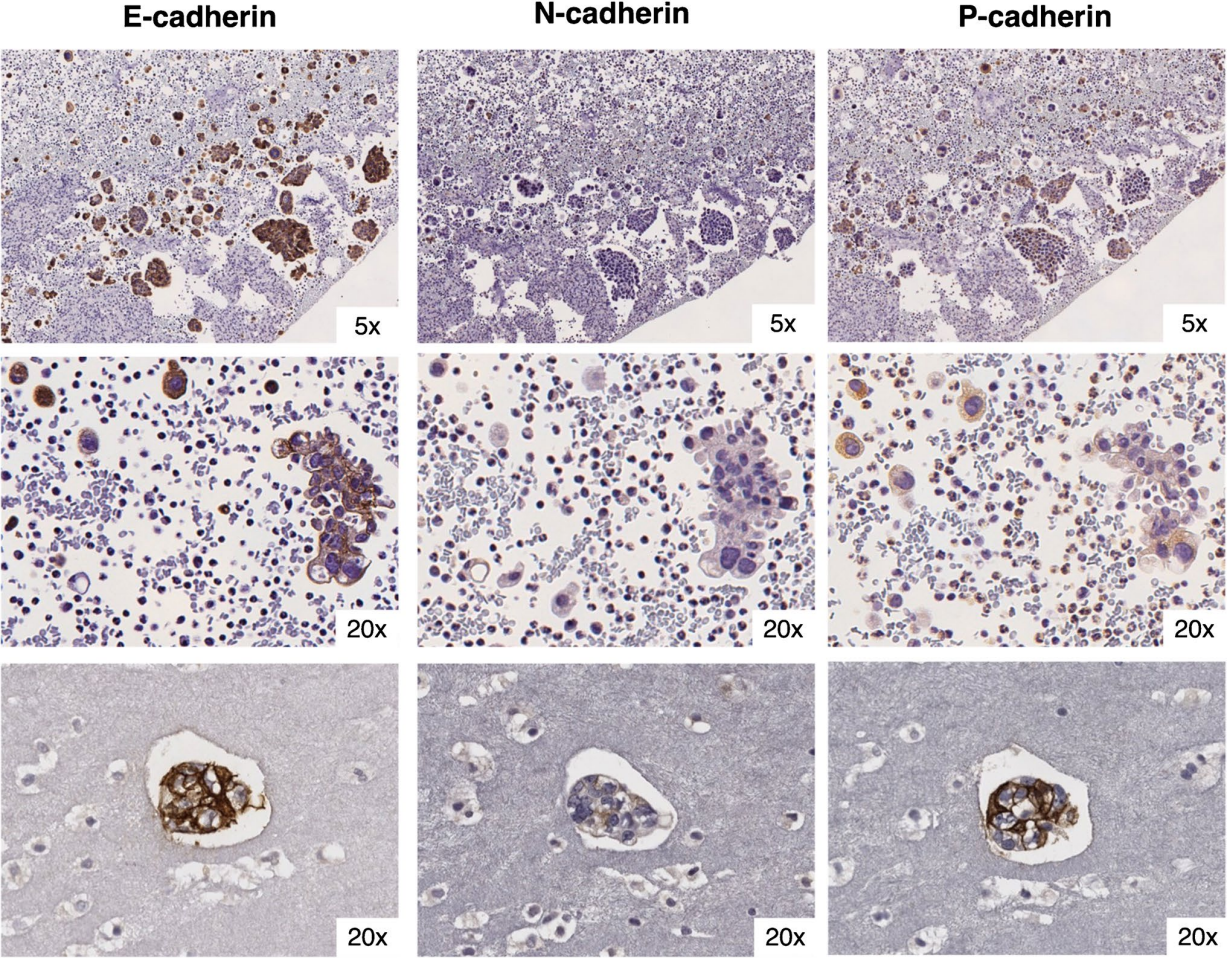
**Cadherins’ expression in malignant ascites from Oporto series**. Representative FFPE cell-blocks images from ascitic fluid of patients with known HGSC stained for E-, N- and P-cadherin. Images display isolated tumour cells and carcinoma cell clusters. These cases pertain to cohort 5, a prospective case series of patients with HGSC and symptomatic malignant ascites from Oporto series (2020-2021). Patients were retrieved from the Pathology department database and after merging with cohort 4 patients’ list, there was one patient in common. Sample collection and processing has been previously described 16. Approximately half of the patients were chemotherapy-naïve at the time of sample collection (52%) and three had known pathogenic alterations in HR genes. Peritoneal fluid was collected for symptom relief and processed into FFPE cell blocks, and cases were included after the presence of HGSC cells in the FFPE cellblocks was confirmed by an experienced cytologist

Transcoelomic spread represents the predominant route of metastasis in HGSC, leading to peritoneal implants and malignant ascites, which are the main causes of morbidity and mortality associated with this histological subtype [24]. Having observed that P-cadherin is upregulated throughout serous carcinogenesis, and since the mesothelium is known to be enriched for the expression of this adhesion molecule, we wondered if P-cadherin could be implicated in the peritoneal dissemination of HGSC cells. To investigate this, we analysed 25 FFPE cell-blocks from malignant peritoneal effusions of patients with HGSC. In most cases (64%), clusters of carcinoma cells predominated over single cells (Fig. 3). In some cases, aggregates containing a mixture of carcinoma, inflammatory and mesothelial cells were identified. E-cadherin proved to be a reliable marker for HGSC cells, being almost universally present on the membrane of carcinoma cells, whether in clusters or isolated. In contrast to its expression in solid tumours, P-cadherin staining was more strongly localized into the cell membrane rather to the cytoplasm and displayed less heterogeneity (Fig. 3). N-cadherin expression was highly heterogeneous, and dot-like staining was not observed. In most cases, E-cadherin and P-cadherin were strongly and diffusely co-expressed at the cell membrane, while N-cadherin displayed a patchy and variable expression pattern. Notably, P-cadherin was significantly overexpressed in ascitic effusions compared to primary tumours or control FTE, while there were no differences compared to precursor lesions (Table 2). In contrast, N-cadherin showed significantly lower expression in ascitic fluid cells compared to the control FTE or precursor lesions, but no differences were observed when comparing with tumours.

In summary, our analysis revealed that N-cadherin expression decreased from normal and pre-malignant to malignant stages, whereas P-cadherin was consistently overexpressed throughout these carcinogenesis steps. Its expression was particularly prominent in precursor lesions and HGSC cells found in ascitic fluid. This suggests that P-cadherin activation may facilitate cell exfoliation and survival in suspension, potentially aiding in their implantation in common sites, such as the ovary and peritoneum.

### P-cadherin overexpression is associated with functional hallmarks of hybrid E/M in vitro

**Fig. 4:**
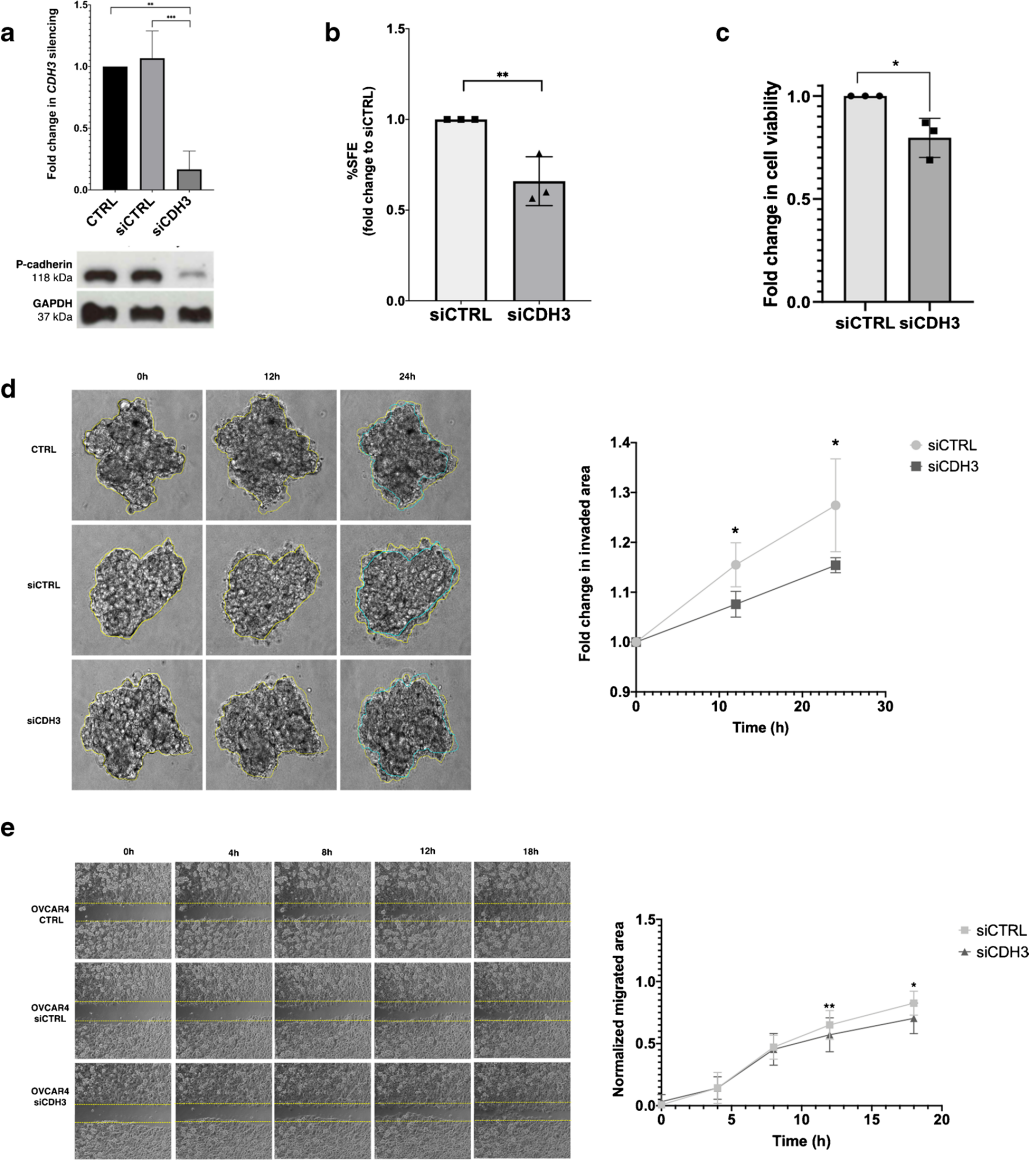
F**unctional assays in OVCAR4 cell line upon CDH3 knockdown with siRNA. a.** Quantification and representative images of Western 48h after CDH3 silencing. **b. **Spheroid-forming assay. Graphic displaying fold change in SFE compared to the control. **c.** Fold change in cell viability and metabolic activity measured by cell viability assay. **d.** Representative experiment of a three-dimensional invasion in collagen assay (right). Graphic on the left displays fold change in invaded area at different timepoints of cell invasion (3 biological replicates). **e.** Representative experiment from a wound healing assay (left). Graphic on the right shows the normalized migrated area at different timepoints of cell migration (3 biological replicates). Only significant differences are highlighted (**** p < 0.0001, *** p < 0.001, ** p < 0.01, * p < 0.05)

Given that P-cadherin has been associated with hybrid E/M features in certain tumour models, we wondered whether P-cadherin overexpression could enhance HGSC dissemination by promoting the acquisition of a hybrid E/M phenotype. Therefore, we evaluated the impact of P-cadherin (encoded by *CDH3*) knockdown on the exhibition of functional hybrid E/M hallmarks in HRP ovarian cell lines. For this, we selected two cell lines expressing high endogenous levels of P-cadherin derived from peritoneal effusions of platinum-resistant HGSC patients (OVCAR3 and OVCAR4) [25, 26] (Fig. S7). We proceeded with the OVCAR4 cell line (Fig. 4a), since OVCAR3 was likely to be HRD [27] and the cadherin expression profile of OVCAR4 more closely recapitulated what we observed in our HGSC case series (Fig. S7). To investigate whether P-cadherin was associated with increased survival under non-adherent conditions, we performed the sphere-forming assay. *CDH3* knockdown led to a significant reduction in sphere-forming efficiency, suggesting that P-cadherin confers an increased resistance to *anoikis* in HGSC cells (Fig. 4b). The role of P-cadherin in collective cell invasion and migration was further evaluated, using the collagen type I invasion and wound healing assays, respectively. P-cadherin knockdown significantly reduced the spheroid expansion (invaded area) and the migratory capacity of cancer cells (Fig. 4d, e). Although cell viability was slightly reduced upon *CDH3* silencing, the small magnitude of this effect suggest that it would not significantly interfere with the observed results (Fig. 4c).

To determine whether these findings were specific to this histological subtype, we performed the same assays on another epithelial ovarian cancer cell line with high endogenous levels of P-cadherin (BG1). This cell line was originally derived from a chemo-naïf poorly differentiated stage III ovarian carcinoma and was reported to have heterogeneous expression of oestrogen and progesterone receptors, and to secrete CA125 in culture [28]. Given that these cells present a mesenchymal phenotype and migrate/invade as single cells, we performed targeted sequencing and an IHC panel to clarify its histologic origin. The BG1 cell line exhibits diffuse PAX8 positivity and heterogeneous WT1 expression (Fig. S8). In addition, our NGS analysis revealed a deleterious *CDH1* mutation, three *PTEN* mutations, and wild-type *TP53* (Table S5). Collectively, these findings strongly support a high-grade endometrioid ovarian carcinoma origin [29]. Upon *CDH3* silencing, there was a significant reduction in the normalized migrated area at 12h and 18h in the wound healing assay, as well as a reduction in the number of invasive cells (Fig. S9). Unlike OVCAR4 cells, no differences were observed in sphere formation or cell viability. Taken together, these findings suggest a link between P-cadherin and hybrid E/M in HGSC-HRP. Furthermore, they also indicate a potential synergistic effect of E-cadherin and P-cadherin in promoting tumour aggressiveness.

## Discussion

In this study, we characterized the immunoexpression patterns of classical cadherins as surrogate markers of EMP dynamics in samples representing the putative stepwise progression of serous carcinogenesis. P-cadherin emerged as the most promising biomarker for the HRP subgroup, as it was significantly overexpressed in precursor lesions and tumours and associated with worst cancer-specific survival, particularly in non-HRD tumours. Here, we demonstrated that a N-to-P-cadherin switch occurs during serous carcinogenesis, which contrasts with previous descriptions of cadherin dynamics in tubo-ovarian carcinoma [30, 31]. This non-canonical switch is characterised by the downregulation of N-cadherin and de novo expression of P-cadherin, while E-cadherin expression remains usually very high throughout all stages, which might reflect a disruption of the normal adhesive properties of the transformed cells. Indeed, N-cadherin is constitutively expressed in the FTE, suggesting that it plays a role in maintaining normal cell–cell adhesion, being therefore an “epithelial-like” marker in this specific context. This contrasts with the classic use of N-cadherin as a mesenchymal marker and highlights the need for context-specific cell-surface markers when evaluating EMP dynamics. Although previous studies describe a canonical E-to-N-cadherin switch in HGSC [30, 31], several reasons may explain the differences in our findings. Firstly, none of the studies has used FTE as the normal counterpart for HGSC carcinogenesis [30, 32]. Secondly, most previous studies included mixed histologic subtypes, relied on outdated WHO classifications, included cases where *BRCA1/2* status was not reported, and did not consistently ensure that sample collection occurred before chemotherapy exposure, limitations that we largely addressed in our study [33–37]. Moreover, it is notable that most tumours co-express the three cadherins in the studies that claim a canonical cadherin switch. For example, Quattrocci et al. described a poor prognostic subgroup with low E-cadherin expression and high N- or P-cadherin expression, but that accounts for only 5% of the cases, while nearly half of the samples exhibited high E-cadherin expression [31]. Interestingly, these authors also found that tumours expressing high levels of the three cadherins showed the worst survival outcomes, suggesting the existence of a hybrid E/M subgroup, corroborating our findings.

Importantly, to our knowledge, this is the first study that evaluates P-cadherin expression during the early steps of serous carcinogenesis. Our data suggest that P-cadherin overexpression may start early in the FTE transformation, in the earliest precursor lesions, such as p53 signatures, STIL or STIC. We also analysed FTE from patients at different risks of developing HGSC, to reduce the bias that could occur when using tissues from the same carcinogenic field. Interestingly, we observed that E- and N-cadherin were generally expressed in control FTE cell membranes regardless the cohort of origin (low or high-risk), while P-cadherin was more frequently expressed in the FTE adjacent to HGSC or STICs, with little or no expression in FTE from patients without malignant lesions. This remarkable finding may reflect an epigenetic field effect and suggests that P-cadherin may be involved in the malignant transformation. Indeed, previous work, using mammary epithelial cells, has shown that P-cadherin is transiently overexpressed during the early stages of EMP activation [38, 39]. Testing the mechanistic impact of P-cadherin overexpression in the FTE with the development of an in vitro model that mimics the stepwise serous tubal carcinogenesis is therefore crucial. Particularly, FTE organoids derived from patients without HRD (low-risk) to model P-cadherin and p53 function could yield important insights in understanding the molecular mechanisms underlying the transformation of some p53 signatures.

In this work, we further observed that tumours with high P-cadherin expression scores were associated with the worst cancer-specific survival. Although this was not found to be an independent prognostic factor in the multivariate analysis, it is aligned with previous findings by other groups [31, 35]. Interestingly, a post-hoc analysis revealed that high P-cadherin expression was significantly associated with shorter cancer-specific survival in non-HRD tumours, whereas this association was not observed in the HRD subgroup. While exploratory in nature, this finding is aligned with our in vitro results, since P-cadherin knockdown reduced sphere-formation, invasion and migration in a HGSC-HRP cell line (OVCAR4), which can be surrogate markers associated to prognosis. These findings establish a link between P-cadherin and hybrid E/M, as observed in other tumour models, where P-cadherin promotes collective cell migration, increased tumour stemness, resistance to *anoikis*, and in vivo tumorigenic capacity [22, 40–46]. Prior studies have also shown that P-cadherin promotes cell–cell aggregation, attachment to the mesothelium, and *anoikis* resistance [47–50], which can be also associated to an enrichment of its expression in ascitic fluids. However, it is noteworthy that certain studies have employed cell lines that are not representative of HGSC or HRP (e.g., SKOV3, HEYA8, and OVCA429). Therefore, P-cadherin’s role in peritoneal dissemination may not be exclusive to the serous subtype but could instead be a feature of aggressive tumours. This warrants further investigation, as we also observed a less aggressive phenotype following *CDH3* knockdown in the BG1 cell line. While originally described as derived from a poorly differentiated ovarian carcinoma [28], our findings provide improved characterization, strongly supporting that BG1 represents a high-grade endometrioid carcinoma of Müllerian origin.

In conclusion, we have demonstrated that a N-to-P-cadherin switch occurs during the malignant transformation of the FTE to HGSC, and that P-cadherin is associated with hybrid E/M functional hallmarks, being therefore a putative marker of these intermediate phenotypes in this tumour model. Building on our key findings, and considering the existing evidence, we speculate that this protein may contribute to the detachment of transformed cells from the FTE by promoting mesenchymal traits, while simultaneously enabling resistance to *anoikis* in the peritoneal cavity through its capacity to maintain cancer cell adhesive clusters, thereby preserving their epithelial properties. Further research in a larger and prospective cohort fully characterized for HRD, as well as dynamic in vivo studies using lineage tracing, is needed to fully characterise the dynamics of EMP and to validate P-cadherin as a new biomarker in HGSC-HRP patients.

## Supplementary Information

Below is the link to the electronic supplementary material.

ESM 1 (PDF 1.44 MB)

ESM 2 (PDF 533 KB)

ESM 3 (PDF 1.43 MB)

ESM 4 (PDF 1.18 MB)

ESM 5 (PDF 65.0 MB)

ESM 6 (PDF 378 KB)

ESM 7 (PDF 8.76 MB)

ESM 8 (PDF 656 KB)

ESM 9 (PDF 50.0 MB)

ESM 10 (PDF 58.8 KB)

ESM 11 (PDF 47.4 KB)

ESM 12 (PDF 42.0 KB)

ESM 13 (PDF 88.0 KB)

ESM 14 (PDF 60.6 KB)
