# Supplementary material for: P-cadherin overexpression is associated with early transformation of the Fallopian tube epithelium and aggressiveness of tubo-ovarian high-grade serous carcinoma
Source: Virchows Arch. 2025 May 5;488(2):309–23. doi: 10.1007/s00428-025-04104-7 (PMC12916920; doi:10.1007/s00428-025-04104-7)
Supplement: Supplementary file 1 — (PDF 1.44 MB) [file 428_2025_4104_MOESM1_ESM.pdf]

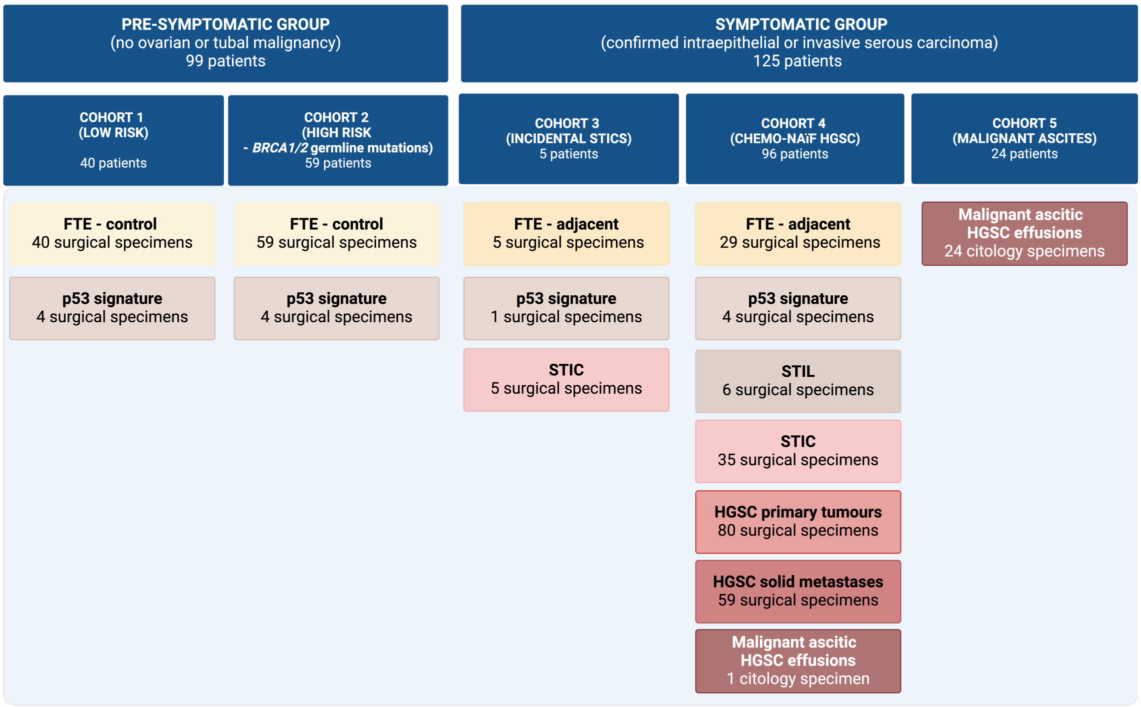

Fig. S1 Samples distribution across the distinct cohorts that compose the Oporto series
