# Supplementary material for: P-cadherin overexpression is associated with early transformation of the Fallopian tube epithelium and aggressiveness of tubo-ovarian high-grade serous carcinoma
Source: Virchows Arch. 2025 May 5;488(2):309–23. doi: 10.1007/s00428-025-04104-7 (PMC12916920; doi:10.1007/s00428-025-04104-7)
Supplement: Supplementary file 2 — (PDF 533 KB) [file 428_2025_4104_MOESM2_ESM.pdf]

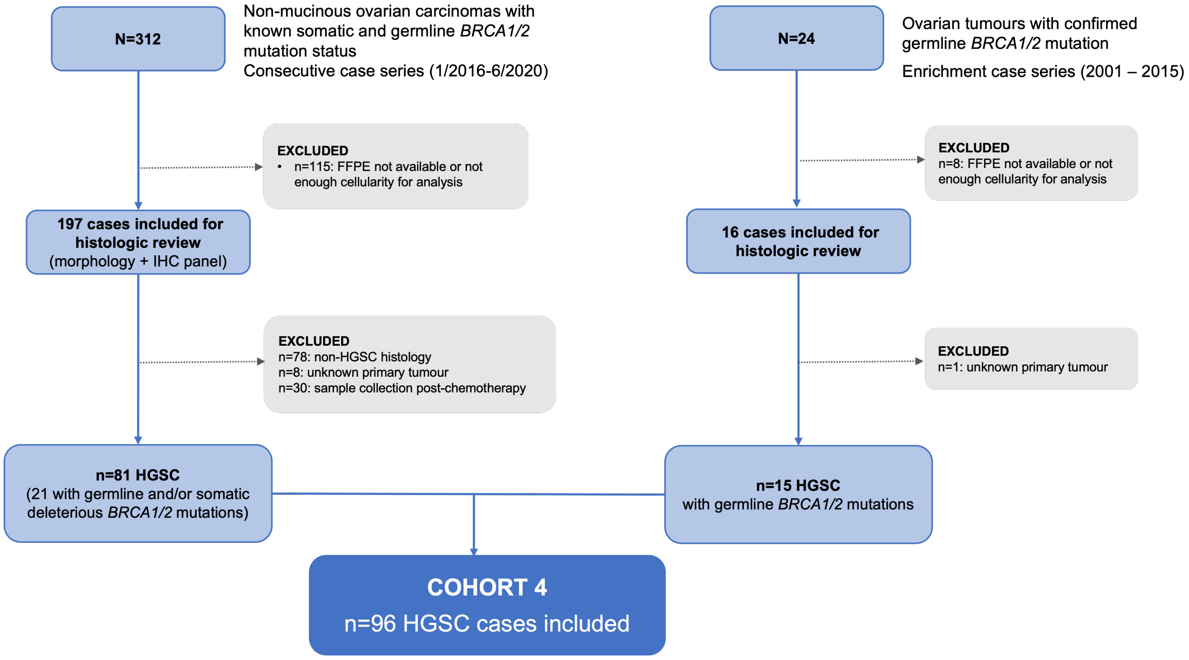

Fig. S2 Flowchart representing the build-up of the Oporto series study cohort 4 (HGSC solid tumours). This cohort consists in a retrospective convenience sample of patients with HGSC whose tumour samples were collected prior to systemic treatment, at the time of surgical staging and/or debulking surgery. Cases were retrieved by merging the Genetics department database (index cases of non-mucinous ovarian cancer tested for *BRCA1/2* deleterious variants) with the Pathology department database (search made using a combination of ICD-O-3.1 topography codes (C48, C56 and C57) and World Health Organization (WHO) 2014 morphology codes for malignant epithelial tumours of the ovary). These lists were merged and patients with diagnosis of non-mucinous ovarian cancer with *BRCA1/2* test were selected for eligibility assessment, which included histology review according to WHO 2014 diagnostic criteria for HGSC, evaluation of tumour representativeness and cellularity. The SEE-FIM protocol was consistently applied to all cases diagnosed since 2012 (82 cases). Staging was reassessed according to 2014 FIGO staging for ovarian, Fallopian tube (FT) and peritoneal cancer. Only cases with a confirmed diagnosis of HGSC whose tissue was collected prior to exposure to chemotherapy were considered eligible. Exclusion criteria were defined as follows: a) HGSC cases in which the primary ovarian, tubal or peritoneal origin was not clear; b) mixed histological subtypes; c) low to very low tumour cellularity, preventing analysis or IHC testing; d) FFPE tissue of FT, ovaries and/or peritoneal metastases not available for analysis; e) cytology specimens or biopsies; f) tissue sample collected after systemic treatment administration. *BRCA1/2* mutation status was used as a surrogate for HRD, as these are the HR genes most frequently displaying deleterious mutation in HGSC. At IPO-Porto, *BRCA1/2* germline mutation screening was performed in the peripheral blood upon a genetic counselling and risk assessment until 2016 and systematic tumour testing for *BRCA1/2* mutation by next generation sequencing was done between 2016-2020. Although a targeted sequencing panel has been implemented in clinical practice since 2020, we only included cases up to 2020 to allow for a minimum of 3 years of follow-up. Subsequently, none of the cases included in this series were tested for other HR mutations, except for those where germline variants were known in the family, which was the case of three patients included with *RAD51D* mutations.
