# Supplementary material for: P-cadherin overexpression is associated with early transformation of the Fallopian tube epithelium and aggressiveness of tubo-ovarian high-grade serous carcinoma
Source: Virchows Arch. 2025 May 5;488(2):309–23. doi: 10.1007/s00428-025-04104-7 (PMC12916920; doi:10.1007/s00428-025-04104-7)
Supplement: Supplementary file 3 — (PDF 1.43 MB) [file 428_2025_4104_MOESM3_ESM.pdf]

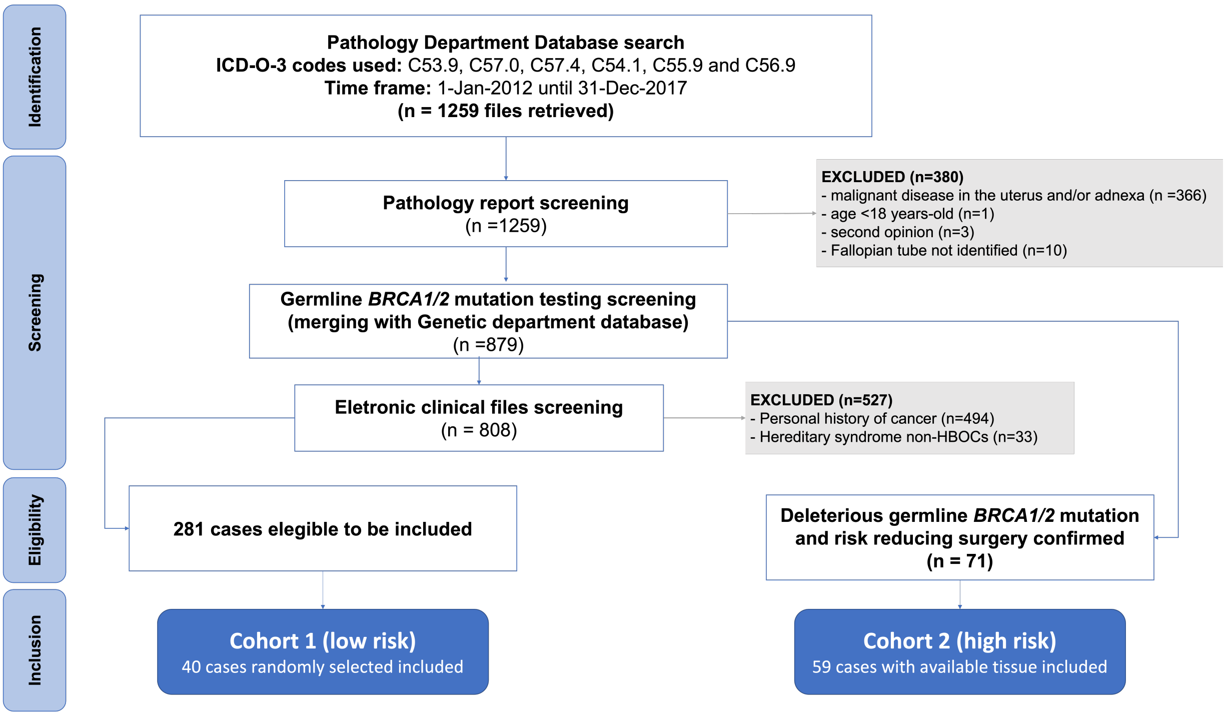

Fig. S3 Flowchart representing the build-up of the Oporto series pre-symptomatic group (cohorts 1 and 2). This group is composed of patients who underwent gynaecological surgery including at least bilateral salpingectomy at IPO-Porto between 2012 and 2017, without malignant lesions identified in the surgical tissue. It was set up as a control group of FTE without malignant involvement of HGSC or STICs, to control for a possible field effect. We selected patients at distinct risk of developing HGSC, stratified by the presence of pathogenic mutations in the *BRCA1/2* genes, to account for this predisposition in our analysis. Cases were retrieved from the Pathology department database using the International Classification of Diseases for Oncology, 3rd edition (ICD-O-3) topographical codes C53.9, C57.0, C57.4, C54.1, C55.9 and C56.9. Cases were then filtered out if there was malignancy in the uterus and adnexa described in the pathological report. The remaining cases were screened manually using the hospital electronic clinical files. One FFPE tissue blocks containing the fimbriated end of one Fallopian tube was randomly selected from each case for further analysis. All samples were processed using the SEE-FIM Protocol (Protocol for Sectioning and Extensively Examining the Fimbriated End of the FT), which has been implemented systematically at IPO-Porto since 2012. This group is composed of two cohorts: a) *Study cohort 1:* Retrospective case series of 40 patients undergoing salpingectomy due to benign gynaecological conditions (e.g., tubal ligation), with no known family history of ovarian / breast cancer and *BRCA1/2* mutation status unknown (low-risk cohort). A random sample was selected from the total of patients retrieved using Randomizer (www.random.org/). b) *Study cohort 2.* Retrospective case series of 59 patients who underwent risk-reducing surgery at IPO-Porto between 2012 and 2017 due to known germline pathogenic *BRCA1/2* mutations (high-risk cohort).
