# Supplementary material for: P-cadherin overexpression is associated with early transformation of the Fallopian tube epithelium and aggressiveness of tubo-ovarian high-grade serous carcinoma
Source: Virchows Arch. 2025 May 5;488(2):309–23. doi: 10.1007/s00428-025-04104-7 (PMC12916920; doi:10.1007/s00428-025-04104-7)
Supplement: Supplementary file 4 — (PDF 1.18 MB) [file 428_2025_4104_MOESM4_ESM.pdf]

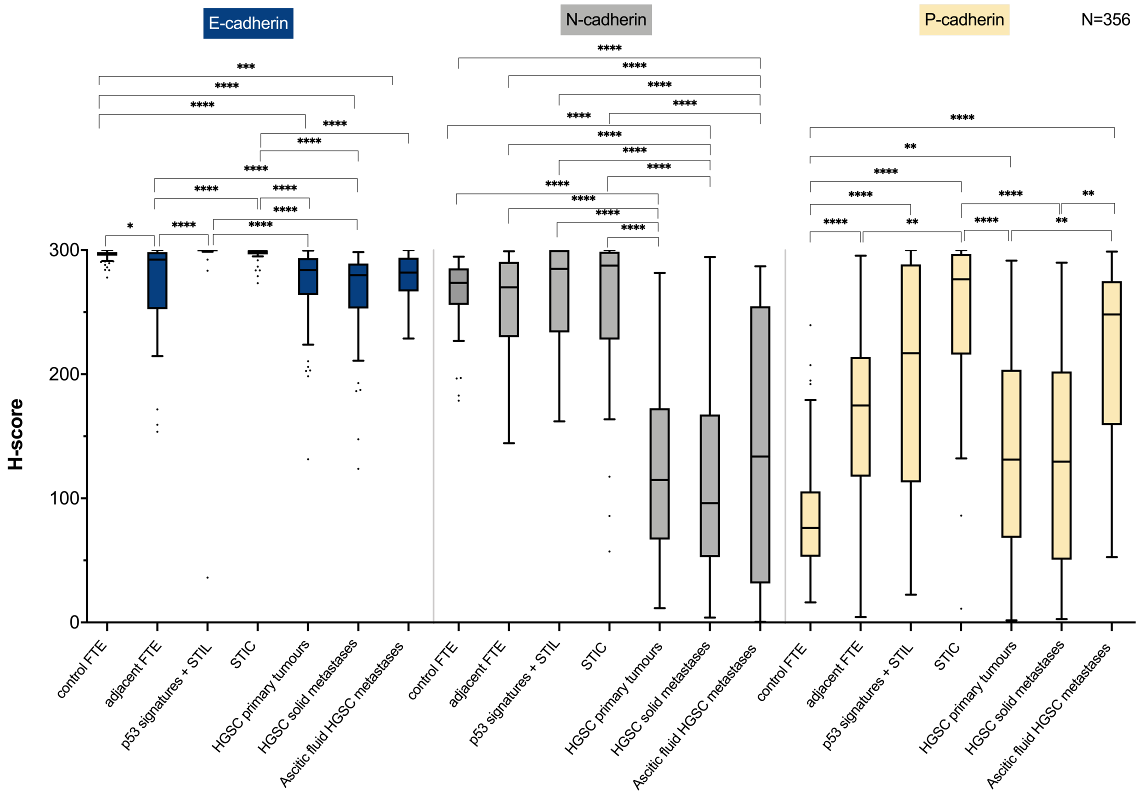

Fig. S4 Cadherin expression in Oporto series Boxplot displaying median and interquartile range H-scores for E-, N- and P-cadherin expression in the different samples included in Oporto series. Comparisons between groups were made using Independent-samples Kruskal Wallis test, with pairwise comparisons. Only significant differences with Bonferroni adjustment for multiple comparisons are shown (**** p<0.0001; ** p<0.01, * p<0.05)
