# Supplementary material for: P-cadherin overexpression is associated with early transformation of the Fallopian tube epithelium and aggressiveness of tubo-ovarian high-grade serous carcinoma
Source: Virchows Arch. 2025 May 5;488(2):309–23. doi: 10.1007/s00428-025-04104-7 (PMC12916920; doi:10.1007/s00428-025-04104-7)
Supplement: Supplementary file 5 — (PDF 65.0 MB) [file 428_2025_4104_MOESM5_ESM.pdf]

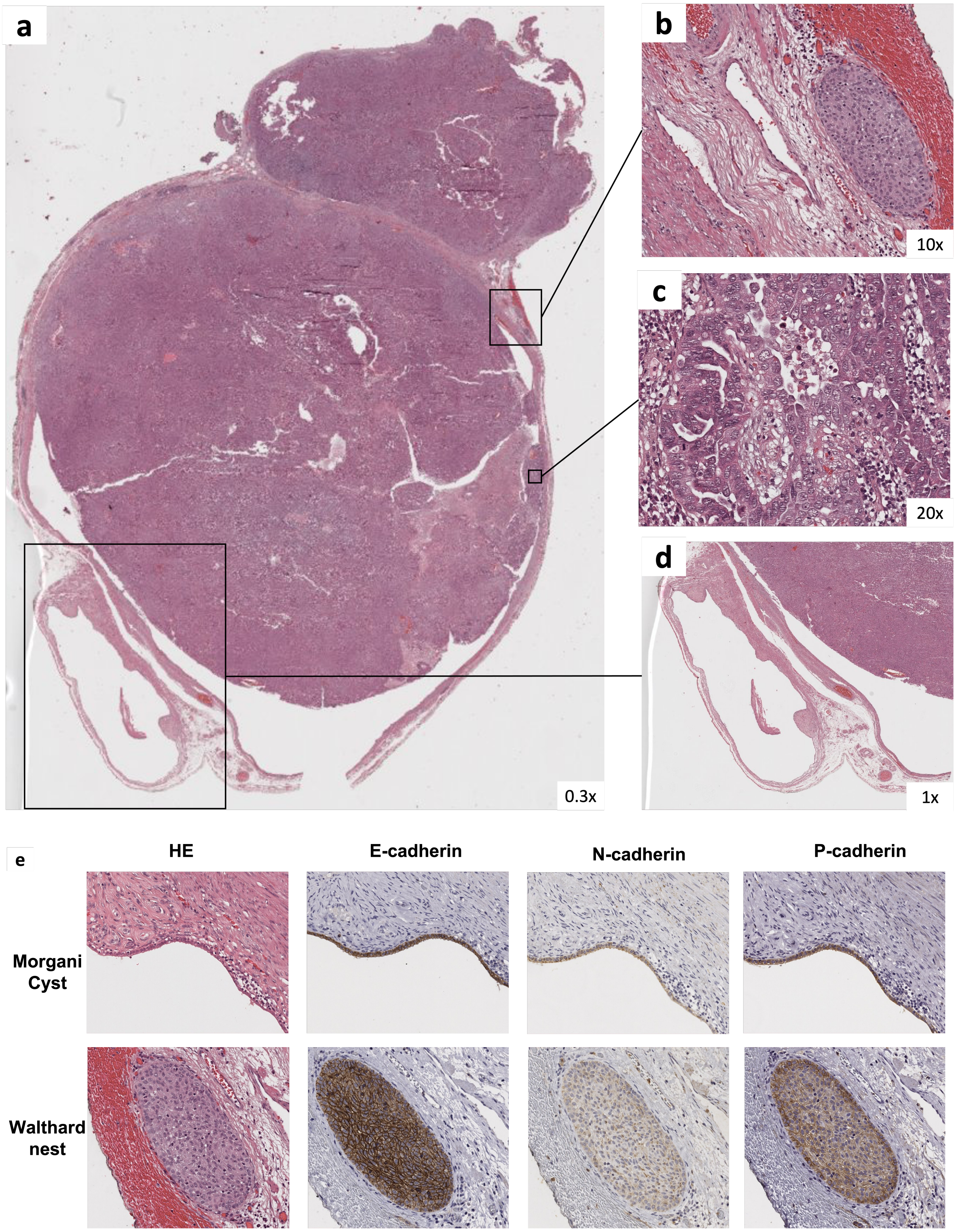

Fig. S5 Cadherin expression in Fallopian tube benign lesions. Histology section displaying an intra-tubar high grade serous carcinoma (a, c) and benign lesions (b – Walthard Nest; d – Morgani Cyst), with distinct patterns of cadherins expression (e, amplification 20x).
