# Supplementary material for: P-cadherin overexpression is associated with early transformation of the Fallopian tube epithelium and aggressiveness of tubo-ovarian high-grade serous carcinoma
Source: Virchows Arch. 2025 May 5;488(2):309–23. doi: 10.1007/s00428-025-04104-7 (PMC12916920; doi:10.1007/s00428-025-04104-7)
Supplement: Supplementary file 6 — (PDF 378 KB) [file 428_2025_4104_MOESM6_ESM.pdf]

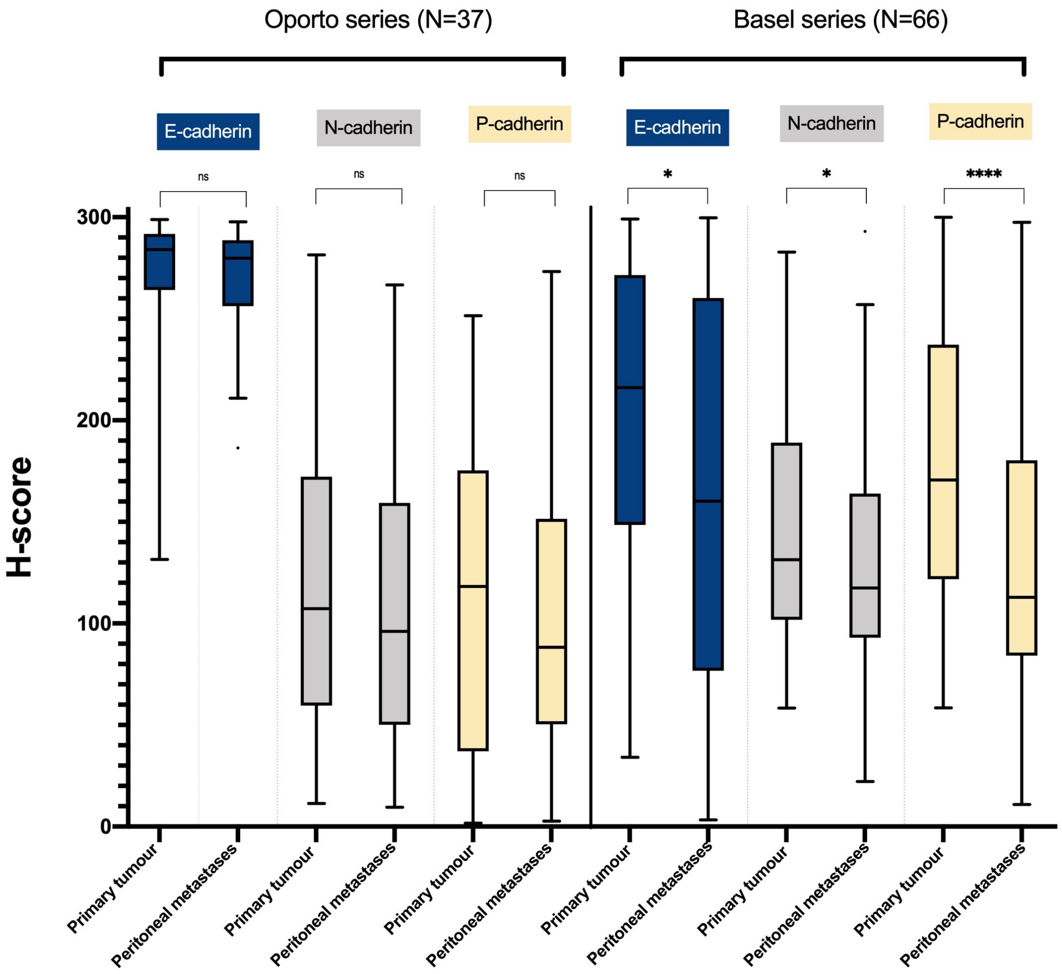

Fig. S6 Cadherins' expression in primary tumours and matched peritoneal metastasis in Oporto and Basel series. Boxplot displaying median and interquartile range H-scores for E-, N- and P-cadherin expression in primary tumours and matched peritoneal metastases. Comparisons were made using Wilcoxon matched-pair signed rank test. Only significant differences with Bonferroni adjustment for multiple comparisons are shown (**** $p<0.0001$; ** $p<0.01$, * $p<0.05$).
