# Supplementary figures and images for: P-cadherin overexpression is associated with early transformation of the Fallopian tube epithelium and aggressiveness of tubo-ovarian high-grade serous carcinoma
Source: Virchows Arch. 2025 May 5;488(2):309–23. doi: 10.1007/s00428-025-04104-7 (PMC12916920; doi:10.1007/s00428-025-04104-7)

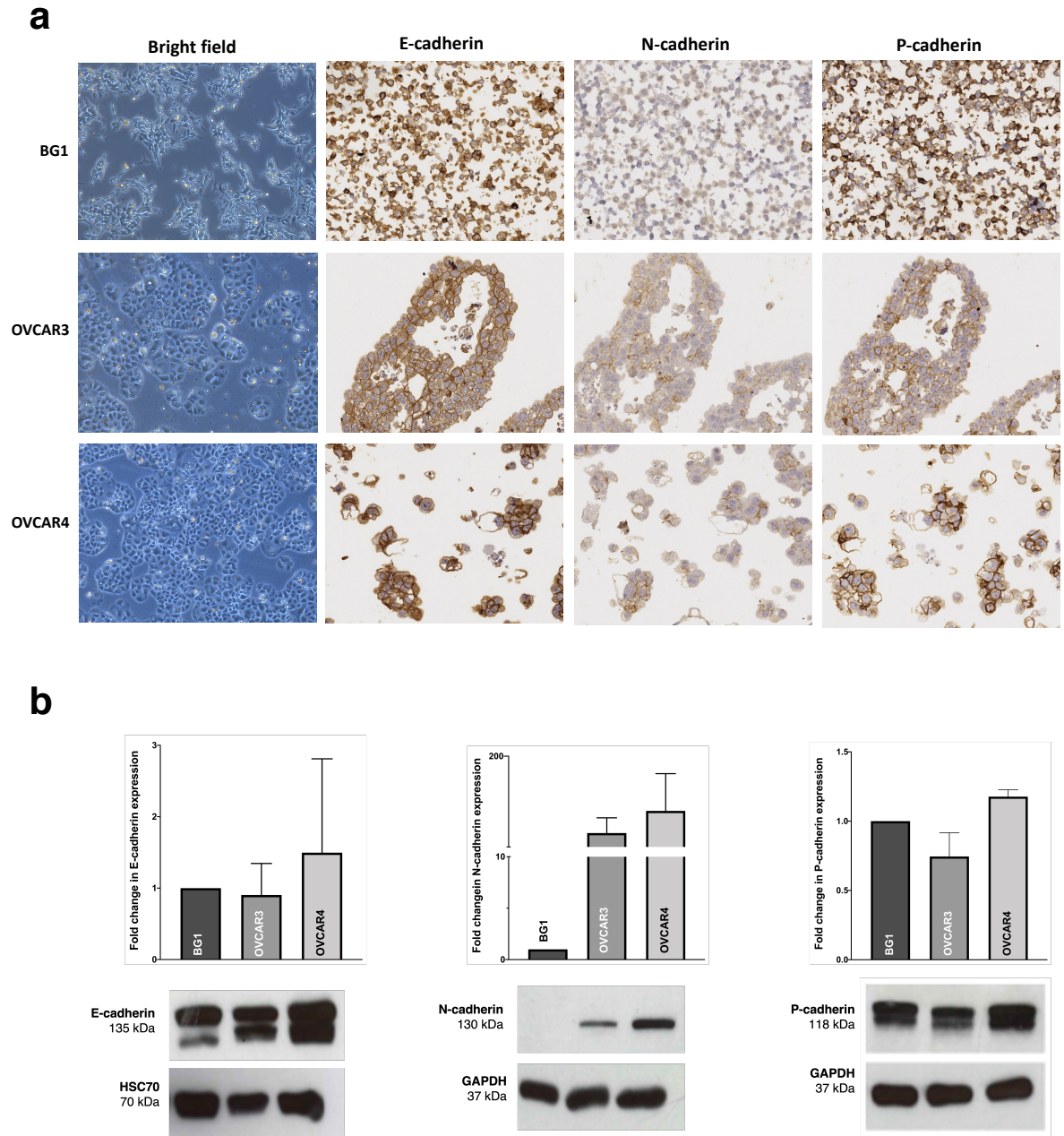

Supplement: Supplementary file 7 — (PDF 8.76 MB) [file 428_2025_4104_MOESM7_ESM.pdf]
