# Supplementary material for: P-cadherin overexpression is associated with early transformation of the Fallopian tube epithelium and aggressiveness of tubo-ovarian high-grade serous carcinoma
Source: Virchows Arch. 2025 May 5;488(2):309–23. doi: 10.1007/s00428-025-04104-7 (PMC12916920; doi:10.1007/s00428-025-04104-7)
Supplement: Supplementary file 8 — (PDF 656 KB) [file 428_2025_4104_MOESM8_ESM.pdf]

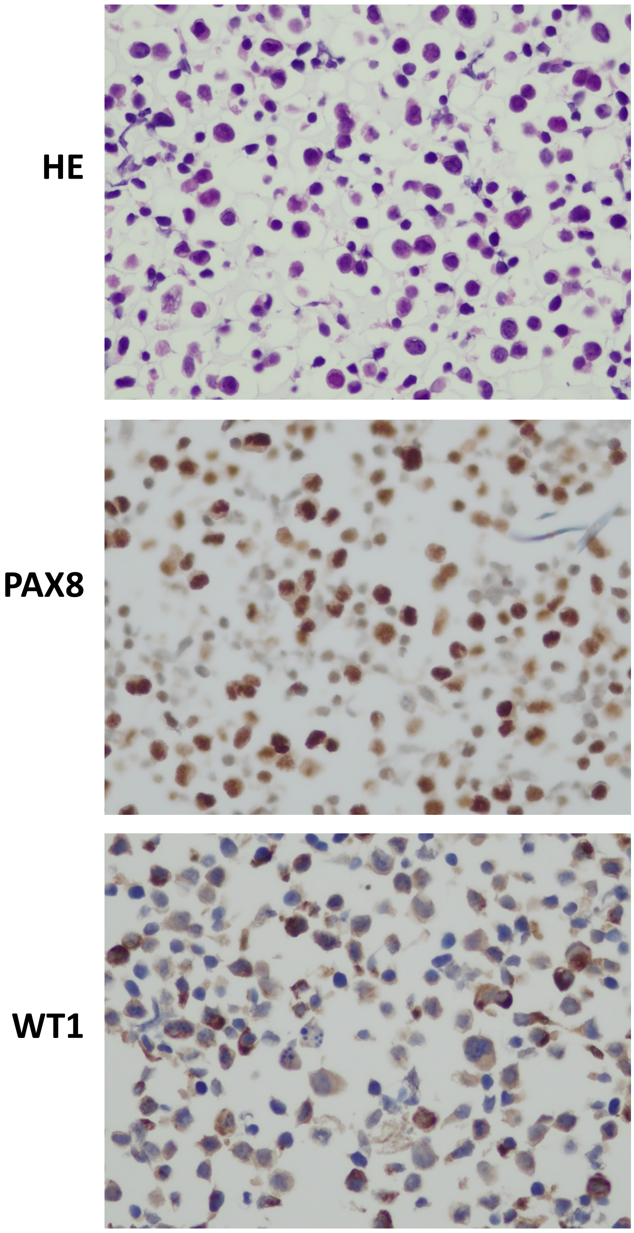

Fig. S8 Immunohistochemical characterization of the BG1 cell line. Panel shows haematoxylin and eosin (HE) staining, PAX8, and WT1 immunoexpression. The BG1 cell line exhibits diffuse PAX8 nuclear staining and heterogeneous WT1 expression (40x amplification).
