# Supplementary material for: P-cadherin overexpression is associated with early transformation of the Fallopian tube epithelium and aggressiveness of tubo-ovarian high-grade serous carcinoma
Source: Virchows Arch. 2025 May 5;488(2):309–23. doi: 10.1007/s00428-025-04104-7 (PMC12916920; doi:10.1007/s00428-025-04104-7)
Supplement: Supplementary file 9 — (PDF 50.0 MB) [file 428_2025_4104_MOESM9_ESM.pdf]

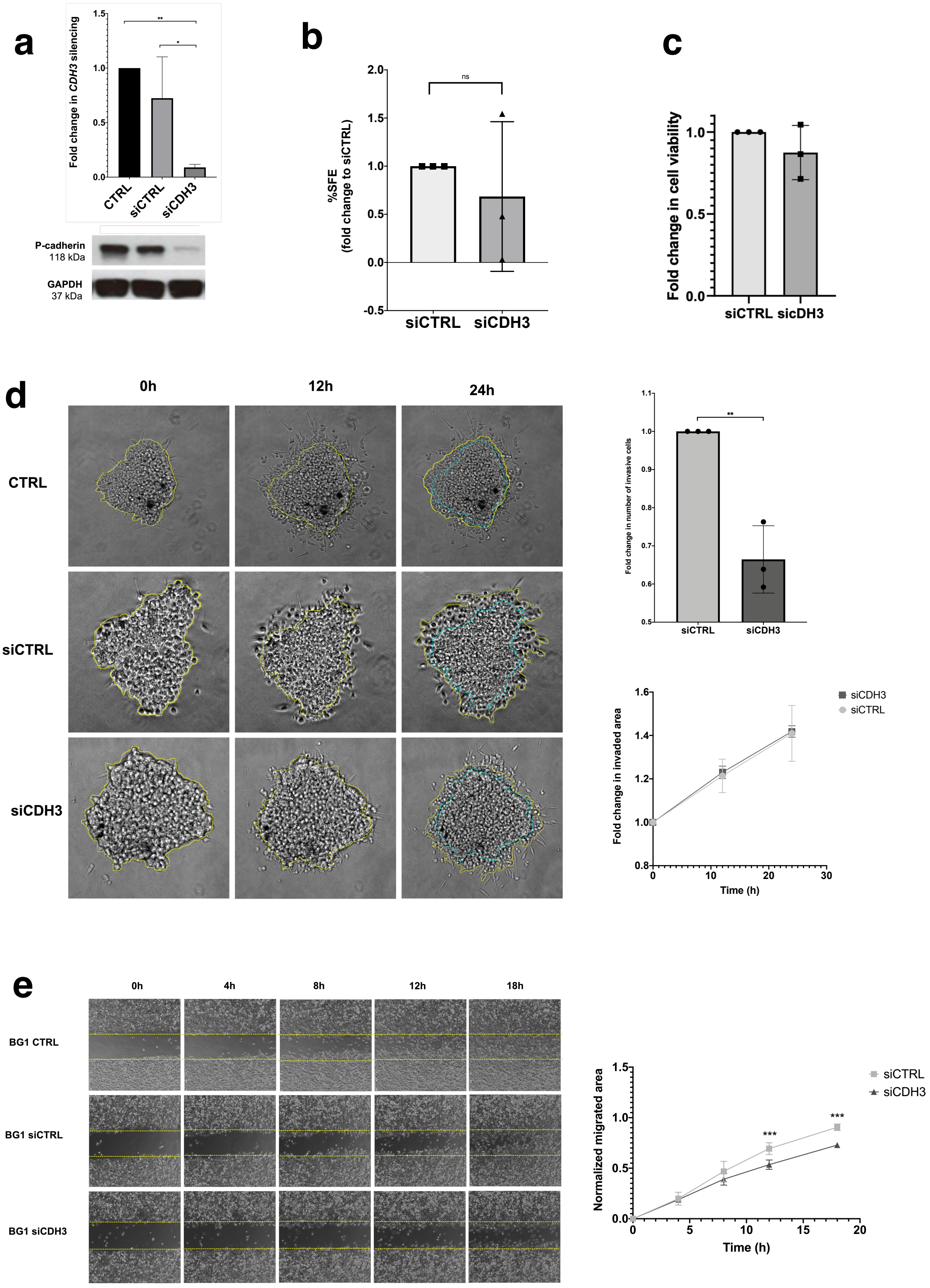

Fig. S9 Functional assays in BG1 cell line upon *CDH3* knockdown with siRNA. a. Quantification and representative images of Western blot, 48h after *CDH3* silencing. **b.** Spheroid-forming assay (3 biological replicates). Graphic displaying fold change in SFE to control. **c.** Fold change in cell viability and metabolic activity measured by a cell viability assay (3 biological replicates). **d.** Representative experiment of a 3D invasion in collagen assay (left). Graphics on the right display fold change in the number of isolated invasive cells (top) and fold change in invaded area at different timepoints of cell invasion (bottom); 3 biological replicates. **e.** Representative experiment from a wound healing assay (left). Graphic on the right shows the normalized migrated area at different timepoints of cell migration (3 biological replicates). siCTRL: control siRNA. SiCDH3: *CDH3* specific siRNA. Only significant differences are highlighted (**** p<0.0001; *** p<0.001, ** p<0.01, * p<0.05).
