# Supplementary material for: P-cadherin overexpression is associated with early transformation of the Fallopian tube epithelium and aggressiveness of tubo-ovarian high-grade serous carcinoma
Source: Virchows Arch. 2025 May 5;488(2):309–23. doi: 10.1007/s00428-025-04104-7 (PMC12916920; doi:10.1007/s00428-025-04104-7)
Supplement: Supplementary file 10 — (PDF 58.8 KB) [file 428_2025_4104_MOESM10_ESM.pdf]

Table S1. Culture and experimental conditions of the ovarian cell lines used

Cell line	*Culture conditions*			*Experimental conditions (cell number per assay)*				
	**Medium**	**Supplementation**	**Atmosphere**	**siRNA transfection**	**Wound Healing assay**	**Collagen type I 3D invasion assay**	**Sphere Formation Assay**	**Cell viability assay**
**OVCAR4**	Roswell Park Memorial Institute (RPMI) 1640 medium GlutaMAX™ with 25nM HEPES	1% antibiotic solution (100 IU/mL penicillin and 100 mg/mL streptomycin, 0.1µm filtered)	Humidified atmosphere with 5% CO_2 at 37ºC	$3.5x10^5$ cells /well (6-well plates)	$2.5x10^5$ cells /well (24-well plate)	1000 cells/well in GravityTRAP™ (PerkinElmer, Shelton, USA) ultra-low attachment 96-well plates	500 cells/cm^2 per well in 6-well plates coated with 1,2% poly-(2-hydroxyethyl methacrylate)/95% ethanol	$3.5x10^4$ cells/well (96-well plates)
**BG1**				$4.5x10^5$ cells /well (6-well plates)	$2.5x10^5$ cells /well (24-well plate)	250 cells/well in GravityTRAP™ (PerkinElmer, Shelton, USA) ultra-low attachment 96-well plates	500 cells/cm^2 per well in 6-well plates coated with 1,2% poly-(2-hydroxyethyl methacrylate)/95% ethanol	$1.5x10^4$ cells/well (96-well plates)
**OVCAR3**				$3.5x10^5$ cells /well (6-well plates)	-	-	-	-
