# Supplementary material for: P-cadherin overexpression is associated with early transformation of the Fallopian tube epithelium and aggressiveness of tubo-ovarian high-grade serous carcinoma
Source: Virchows Arch. 2025 May 5;488(2):309–23. doi: 10.1007/s00428-025-04104-7 (PMC12916920; doi:10.1007/s00428-025-04104-7)
Supplement: Supplementary file 11 — (PDF 47.4 KB) [file 428_2025_4104_MOESM11_ESM.pdf]

Table S2. List of antibodies

Reagent / Antibody	Source	Catalog number	Dilution	Species reactivity
**E-cadherin**	Cell Signalling, USA	clone 24E10	Imunohistochemisty (IHC): 1:100; Western Blot (WB): 1:1000	rabbit
**N-cadherin**	ThermoFisher, USA	clone 3B9	IHC: 1:100; WB: 1:1000	mouse
**P-cadherin**	BD Transduction, USA	clone 56, BD	IHC: 1:50; WB: 1:1000	mouse
**p53**	DAKO, USA	clone DO-7	IHC: 1:1200; WB: 1:1000	rabbit
**ki67**	DAKO, USA	clone MIB-1	IHC: 1:150	mouse
**PAX8**	Roche, Switzerland	clone MRQ-50	IHC: ready to use	mouse
**GAPDH**	Santa Cruz Biotechnology Inc, USA	clone sc-47724	WB: 1:1000	mouse
**HSP70**	Santa Cruz Biotechnology Inc, USA	clone sc-7298	WB 1:1000	mouse
**Alpha-tubulin**	Sigma-Aldrich, USA	clone DM1A	WB 1:10000	mouse
**Mouse anti-rabbit IgG-HRP**	Santa Cruz Biotechnology Inc, USA	clone sc-2357	WB 1:2000	mouse
**Anti-mouse IgGκ- HRP**	Santa Cruz Biotechnology Inc, USA	clone sc-516102	WB 1:2000	rabbit
