# Supplementary material for: P-cadherin overexpression is associated with early transformation of the Fallopian tube epithelium and aggressiveness of tubo-ovarian high-grade serous carcinoma
Source: Virchows Arch. 2025 May 5;488(2):309–23. doi: 10.1007/s00428-025-04104-7 (PMC12916920; doi:10.1007/s00428-025-04104-7)
Supplement: Supplementary file 12 — (PDF 42.0 KB) [file 428_2025_4104_MOESM12_ESM.pdf]

Table S3. List of reagents

Reagent	Source
siRNA specific for *CDH3* (Hs_CDH3_6), final concentration 50nM	Qiagen, Germany
siRNA negative control	Qiagen, Germany
Lipofectamine ™ 2000 transfection reagent	Invitrogen, USA
PrestoBlue™ reagent	ThermoFisher, USA
Trypsin-EDTA Gibco™	ThermoFisher, USA
Rat tail collagen type I	Merck KGaA, Darmstadt, Germany
Roswell Park Memorial Institute (RPMI) 1640 medium, GlutaMAX™ supplemented with 25nM HEPES Gibco™	ThermoFisher, USA
Heat-inactivated foetal bovine serum	Cytiva, Germany
Penicillin-Streptomycin	Sigma-Aldrich, USA
poly-(2-hydroxyethyl methacrylate)	Sigma-Aldrich, USA
Phenol red-free DMEM/F12 medium	Invitrogen, USA
Human EGF	Sigma-Aldrich, USA
B27	Invitrogen, USA
Insulin	Sigma-Aldrich, USA
Triton	Sigma-Aldrich, USA
IGEPAL ® CA-630	Sigma-Aldrich, USA
Protease inhibitor cocktail	Roche Diagnostics Gmbh, Mannheim, Germany
Phosphatase inhibitor cocktail	Sigma-Aldrich, USA
Bio-Rad protein assay	BioIRad, Richmond, CA, USA
ECL Chemiluminescence detection reagent	Amersham Pharmacia Biotech, Piscataway, NJ, USA
