# Supplementary material for: P-cadherin overexpression is associated with early transformation of the Fallopian tube epithelium and aggressiveness of tubo-ovarian high-grade serous carcinoma
Source: Virchows Arch. 2025 May 5;488(2):309–23. doi: 10.1007/s00428-025-04104-7 (PMC12916920; doi:10.1007/s00428-025-04104-7)
Supplement: Supplementary file 13 — (PDF 88.0 KB) [file 428_2025_4104_MOESM13_ESM.pdf]

Table S4. Correlation of clinicopathologic features traditionally associated with prognosis in HGSC with cadherin expression

		E-CADHERIN			N-CADHERIN			P-CADHERIN		
		**Low**	**High**	Pearson X^2	**Low**	**High**	Pearson X^2	**Low**	**High**	Pearson X^2
**Age**	<58	20	28	p>0.05	25	23	p>0.05	25	23	p>0.05
	>= 58	28	20		22	26		21	27	
**Cytorreduction**	Yes	37	38	p>0.05	38	37	p>0.05	39	36	p>0.05
	No	2	3		1	4		1	4	
**Completeness of cytoreductive surgery** [a]	Complete	12	20	p>0.05	18	14	p>0.05	18	14	p>0.05
	Incomplete	11	14		11	14		12	13	
**HR status**	HRD	15	16	p>0.05	15	16	p>0.05	16	15	p>0.05
	Non-HRD	24	25		24	25		24	25	
**g*BRCA1/2* status**	mutated	11	12	p>0.05	13	10	p>0.05	12	11	p>0.05
	wildtype	28	29		26	31		28	29	
**FIGO 2014 staging**	I-II	7	7	p>0.05	6	8	p>0.05	8	6	p>0.05
	III-IV	32	33		32	33		32	33	
**Neoadjvant chemotherapy**	Yes	8	12	p>0.05	7	13	p>0.05	8	12	p>0.05
	No	31	29		32	28		32	28	

a. Completeness of cytoreductive surgery: surgical debulking for HGSOC was considered incomplete if macroscopic residual disease was greater than 1 cm (Ledermann, J.A., et al., *ESGO-ESMO-ESP consensus conference recommendations on ovarian cancer: pathology and molecular biology and early, advanced and recurrent disease.* Annals of Oncology, 2024)
