# Supplementary material for: P-cadherin overexpression is associated with early transformation of the Fallopian tube epithelium and aggressiveness of tubo-ovarian high-grade serous carcinoma
Source: Virchows Arch. 2025 May 5;488(2):309–23. doi: 10.1007/s00428-025-04104-7 (PMC12916920; doi:10.1007/s00428-025-04104-7)
Supplement: Supplementary file 14 — (PDF 60.6 KB) [file 428_2025_4104_MOESM14_ESM.pdf]

Table S5. Targeted sequencing panel performed in BG1 cell line

Gene	Transcript	HGVS coding	Predicted protein	VAF (%)	ClinVar classification (ClinVar ID)
CDH1	NM_004360.5	**c.1942G>T**	p.(Glu648Ter)	36	Pathogenic (463735)
PTEN	NM_000314.8	**c.44G>A**	p.(Arg15Lys)	49	Likely Pathogenic (404147)
PTEN	NM_000314.84	**c.517C>T**	p.(Arg173Cys)	41	Pathogenic (189500)
PTEN	NM_000314.84	**c.389G>A**	p.(Arg130Gln)	41	Pathogenic (7829)
